# Comparative transcriptome profiling of low light tolerant and sensitive rice varieties induced by low light stress at active tillering stage

**DOI:** 10.1038/s41598-019-42170-5

**Published:** 2019-04-08

**Authors:** Sudhanshu Sekhar, Darshan Panda, Jitendra Kumar, Niharika Mohanty, Monalisha Biswal, Mirza J. Baig, Awadhesh Kumar, Ngangkham Umakanta, Sangamitra Samantaray, Sharat K. Pradhan, Birendra P. Shaw, Padmini Swain, Lambodar Behera

**Affiliations:** 1 ICAR-National Rice Research Institute, Cuttack, Odisha 753006 India; 2 Institute of Life Sciences, Nalco Square, Bhubaneswar, Odisha 751023 India

## Abstract

Low light intensity is a great limitation for grain yield and quality in rice. However, yield is not significantly reduced in low light tolerant rice varieties. The work therefore planned for comparative transcriptome profiling under low light stress to decipher the genes involved and molecular mechanism of low light tolerance in rice. At active tillering stage, 50% low light exposure for 1 day, 3 days and 5 days were given to Swarnaprabha (low light tolerant) and IR8 (low light sensitive) rice varieties. Illumina (HiSeq) platform was used for transcriptome sequencing. A total of 6,652 and 12,042 genes were differentially expressed due to low light intensity in Swarnaprabha and IR8, respectively as compared to control. CAB, LRP, SBPase, MT15, TF PCL1 and Photosystem I & II complex related gene expressions were mostly increased in Swarnaprabha upon longer duration of low light exposure which was not found in IR8 as compared to control. Their expressions were validated by qRT-PCR. Overall study suggested that the maintenance of grain yield in the tolerant variety under low light might be results of accelerated expression of the genes which enable the plant to keep the photosynthetic processes moving at the same pace even under low light.

## Introduction

Light is one of the important natural resources for primary growth and production in plants. It is required not only for photosynthesis but also for overall development of plants including physiological processes like photo morphogenesis and reproductive stage development^1–3^. The importance of light for plants is so much that even low light intensity is considered as one of the important abiotic stresses because it affects photosynthesis resulting in decrease in yield potential. About 40 to 50% yield loss is mostly experienced in rice due to low light intensity during wet season in India and South East Asian countries where decrease in irradiation is experienced up to 40 to 60%^4,5^. Low light affects all the stages of rice growth. It significantly reduces the number of tillers and panicles at vegetative stage^4^ while it causes reduction in spikelet number, grain weight and grain quality at the reproductive stage^6–8^. Besides, it affects plant height, shoot and root growth^9^ Low light causes varieties of biochemical and physiological disturbances in plants. It is established that low light stress affects physiological metabolism of rice plants such as activities of antioxidant enzymes in leaves and key enzymes involved in starch synthesis in grains as well as the translocations of carbohydrate from source to sink cells^10^. These studies showed that low light is a serious problem in rice cultivation.

Despite the prevalent problem of low light intensity associated with rice crop, breeders have been successful in developing rice varieties tolerant to low light stress. Swarnaprabha (an *indica* rice variety) is consistent in yield under normal as well as low light condition^5,11,12^. The tolerance of Swarnaprabha to low light is because of its ability to change its physiological, biochemical and molecular level activities as per the requirement necessitated by the available light intensity^13^. Earlier research results indicates that the varieties that are tolerant to low light contain higher chlorophyll b in leaves and maintain a lower chlorophyll a/b ratio when subjected to low light as compared to normal light^10^. Furthermore, low light tolerant varieties (such as Swarnaprabha) maintain their carbohydrate production levels by maintaining an efficient photosynthetic rate even under low light and possess efficient antioxidant capacity, which in turn is achieved by maintaining higher level of chlorophyll and antioxidant enzymes compared to the low-light sensitive varieties^10,14,15^. The research work undergoes in the field at molecular level was not very much done and genes involved in low-light tolerance in rice is however, poorly understood.

In the present study, we have used comparative transcriptomic approach to decipher the genes associated with low-light tolerance in rice considering that physiological functions are largely reflection of changes at the transcript level. In this process, transcriptome of two rice varieties, Swarnaprabha, a low light tolerant and IR8, a low light sensitive^16^ were compared after exposing them to 50% less light intensity for various duration at active tillering stage with respect to their control, i.e. the plants receiving normal light intensity. We detected several important genes those differentially expressed were belong to photosynthesis and components of the light harvesting complex in the low-light tolerant variety, Swarnaprabha compared to the low-light sensitive variety, IR8 upon low-light treatment. This is the first report on molecular mechanism of low light intensity tolerance in rice.

## Results

### Photosynthetic active radiation (PAR) and Chlorophyll content and Chlorophyll a/b ratio

**Figure 1:**
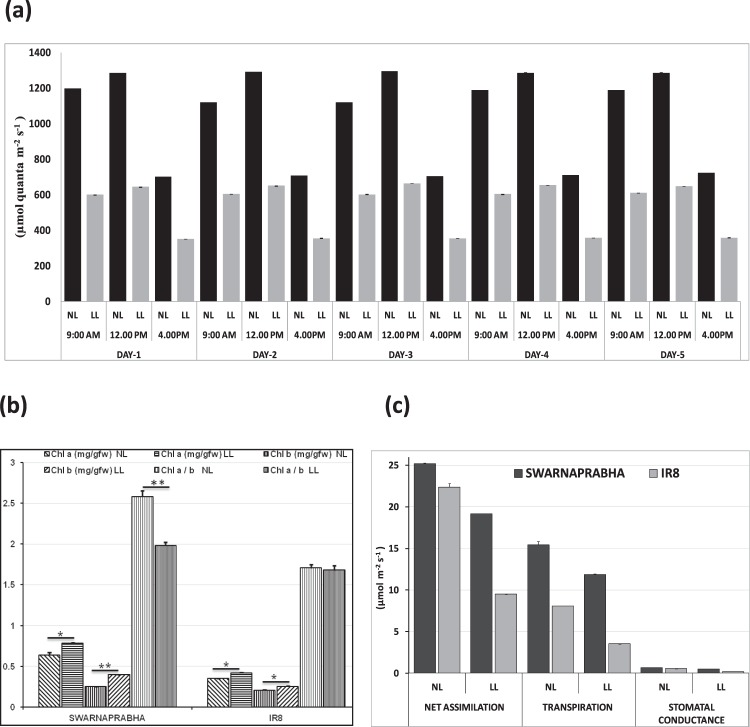
(**a**) Spatiotemporal distribution of Photosynthetic active radiation (PAR) above the canopy of both Swarnaprabha and IR8 under normal (NL) and low light (LL) conditions (Data are means ± SD of n = 5 experiments). (**b**) Chla content P < 0.05 (t-test for mean at α = 0.05), Chlb content in Swarnaprabha at the level of P < 0.01 and Chl a/b ratio (t-test for mean data α = 0.01), data were taken after 5 days low light treated samples for both Swarnaprabha (low light tolerant) and IR8 (low light sensitive). (**c**) Photosynthetic parameter measurements: Net assimilation, stomatal conductance and transpiration measurements were recorded for both open system and treatment with a CO_2_ concentration of 380 µmol l^−1^ under available light condition for Swarnaprabha. LL- low light, N-normal Light. An average value was calculated from five flag leaves from each replicate.

Measurement of PAR under low light and normal light intensity above canopy of both the varieties Swarnaprabha and IR8 was recorded (Fig. 1a) during three times of the day (9.00 am, 12.00 pm and 4.00 pm of Indian Standard Time (IST), UTC + 05:30) for 1 day, 2 days, 3 days, 4 days and 5 days after low light treatment to confirm the low light stress and normal light intensity. After 5 days of low light treatment, chlorophyll a and chlorophyll b content of both the varieties were recorded. The chlorophyll a content was significantly increased in both the varieties under low light at P < 0.05 (t-test for mean at α = 0.05), but chlorophyll b content was significantly increased in Swarnaprabha at P < 0.01, and therefore the chlorophyll a/b ratio (Fig. 1b) was significantly decreased (P < 0.01, t-test for mean at α = 0.01) in Swarnaprabha. However, chlorophyll b content was not significantly increased in IR8, indicating that low light tolerant variety Swarnaprabha increases its chlorophyll b content to greater extent under low light in order to capture solar energy as per requirement and maintain the photosynthetic process even under low light condition. Further, physiological parameter such as net assimilation, transpiration and stomatal conductance were estimated in both the varieties under normal and low light condition (Fig. 1c). Results indicated that all physiological parameters have higher values in low light tolerant variety, Swarnaprabha compared to low light sensitive variety, IR8.

### Transcriptome data assembly and analysis

**Figure 2:**
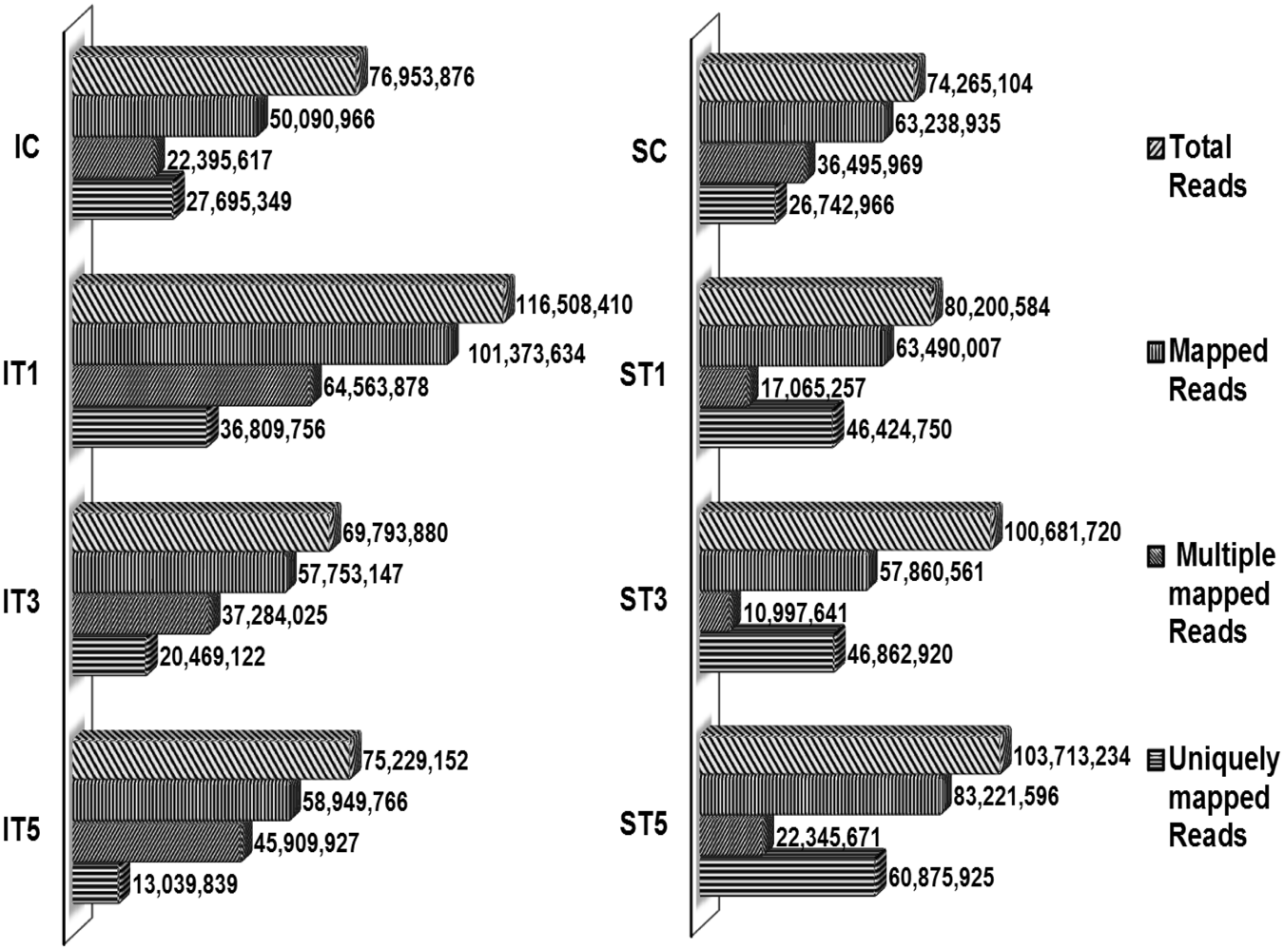
Alignment statistics of transcriptome of treated and control samples of rice varieties, Swarnaprabha (low light tolerant) and IR8 (low light sensitive) with reference genome, identified through Illumina sequencing using TopHat 2 software. IC-IR8 control; IT1-IR8 for 1 day low light treated; IT3-IR8 for 3 days low light treated; IT5-IR8 for 5 days low light treated; SC- Swarnaprabha control; ST1- Swarnaprabha for 1 day low light treated; ST3- Swarnaprabha for 3 days low light treated; ST5-Swarnaprabha for 5 days low light treated.

In order to get a deeper insight into the low light stress responses of tolerant (Swarnaprabha) and sensitive (IR8) rice varieties, a total eight libraries were prepared and sequenced using Illumina HiSeq 2500 platform. The raw reads and clean reads of transcriptome data and their statistical analysis were listed in the Table 1. The quality control of raw reads was assessed through the analysis of sequence quality, GC content, the presence of adaptors and duplicated reads in order to detect sequencing errors. Phred quality score (Q-score) was used to assess the accuracy of a sequence. The value of Q20 was above 96% for all samples while Q30 value was above 91% (Table 1) indicating high accuracy of the sequencing. GC content of all samples varied from 53.05% to 55.87% with an average of 55.07%. A total of 372.2 million clean reads for Swarnaprabha and 351.4 million clean reads for IR8 were obtained with an average of 93.05, 87.85 million clean reads per sample, respectively. The clean reads of each of the sample was aligned individually to *Oryza sativa japonica* (Ensembl IRGSP-1.0) reference genome using TopHat 2 (A spliced read mapper for RNA-Seq). More than 267.8 million clean reads of Swarnaprabha and more than 268.1 million clean reads of IR8 were mapped to the reference genome. Total number of clean reads mapped and percentage of mapping of clean reads for each sample were listed in Table 2. Furthermore, multiple mapped read and uniquely mapped reads were calculated from the total clean reads which were shown as alignment statistics of mapping for Swarnaprabha and IR8 (Fig. 2). Fragments per kilobase of transcript per million mapped reads (FPKM) and total read count of differentially expressed gene were listed in Supplementary Table S2a and S2b, respectively.

**Table 1:** Basic statistics of total raw and clean reads of transcriptome sequencing for control and low light treated samples of rice varieties Swarnaprabha (low light tolerant) and IR8 (low light sensitive).

Sample	Raw Reads	Clean Reads	Raw Base	Clean Base	Effective rate	Error Rate	Q20 (%)	Q30 (%)	GC-Content (%)
SC	77344312	74265104	11.6	11.14	96.02	0.02	97.28	93.06	54.72
ST1	83612018	80200584	12.54	12.03	95.92	0.03	97.16	92.84	54.65
ST3	104021776	100681720	15.6	15.1	96.79	0.03	96.12	91.06	55.3
ST5	107903734	103713234	16.19	15.56	96.12	0.03	97.16	92.79	55.81
IC	79761464	76953876	11.96	11.54	96.48	0.03	96.89	92.5	53.05
IT1	121481620	116508410	18.22	17.48	95.91	0.03	97.36	93.16	55.87
IT3	72504310	69793880	10.88	10.47	96.26	0.03	96.48	91.2	55.86
IT5	77675456	75229152	11.65	11.28	96.85	0.03	97.06	92.63	55.28
**Total**	**724304690**	**697345960**	**108**.**64**	**104**.**6**	**770**.**35**	**0**.**23**	**775**.**51**	**739**.**24**	**440**.**54**
**Mean**	**90538086**.**25**	**87168245**.**00**	**13**.**58**	**13**.**08**	**96**.**29**	**0**.**03**	**96**.**94**	**92**.**41**	**55**.**07**

SC- Swarnaprabha control, ST1- Swarnaprabha for 1 day low light treated; ST3- Swarnaprabha for 3 days low light treated; ST5- Swarnaprabha for 5 days low light treated; IC- IR8 control; IT1- IR8 for 1 day low light treated; IT3- IR8 for 3 days low light treated; IT5- IR8 for 5 days low light treated.

**Table 2:** Mapping statistics of clean reads of RNA sequencing data to reference rice genome in control and low light treated samples of rice varieties Swarnaprabha (low light tolerant) and IR8 (low light sensitive).

Sample name	Total clean reads	No of clean reads mapped to genome	% of all cleaned reads aligned to genome
SC	74265104	63238935	85.15
ST1	80200584	63490007	79.16
ST3	100681720	57860561	57.47
ST5	103713234	83221596	80.24
IC	76953876	50090966	65.09
IT1	116508410	101373634	87.01
IT3	69793880	57753147	82.75
IT5	75229152	58949766	78.36
**Total**	**697345960**	**535978612**	**615**.**23**
**Mean**	**87168245**	**66997326**.**5**	**76**.**90**

SC- Swarnaprabha control, ST1- Swarnaprabha for 1 day low light treated; ST3- Swarnaprabha for 3 days low light treated; ST5- Swarnaprabha for 5 days low light treated; IC- IR8 control; IT1- IR8 for 1 day low light treated; IT3- IR8 for 3 days low light treated; IT5- IR8 for 5 days low light treated sample.

### Influence of low light treatment on gene expression in Swarnaprabha and IR8

**Figure 3:**
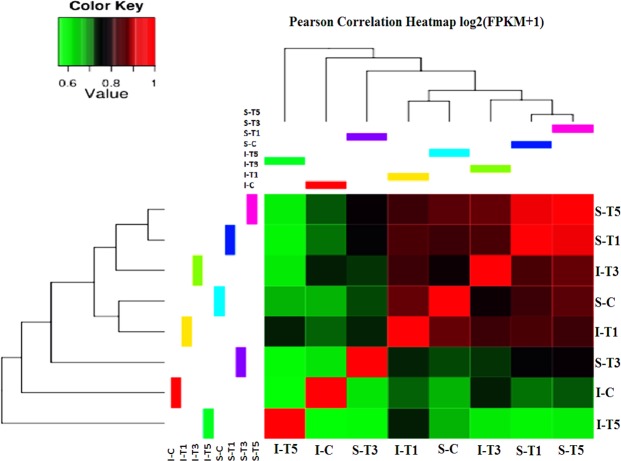
Heatmap for the Pearson Correlation Coefficient between samples of Swarnaprabha and IR8. The closer the correlation coefficient is to 1, the greater the similarity of the samples. Each coefficient in the correlation matrix is represented as a square, with the colour of the square representing the amount of correlation. The colour scale used is green to red, with green representing 0, red representing 1. IC-IR8 control; IT1-IR8 for 1 day low light treated; IT3-IR8 for 3 days low light treated; IT5-IR8 for 5 days low light treated; SC- Swarnaprabha control; ST1- Swarnaprabha for 1 day low light treated; ST3- Swarnaprabha for 3 days low light treated; ST5-Swarnaprabha for 5 days low light treated.

**Figure 4:**
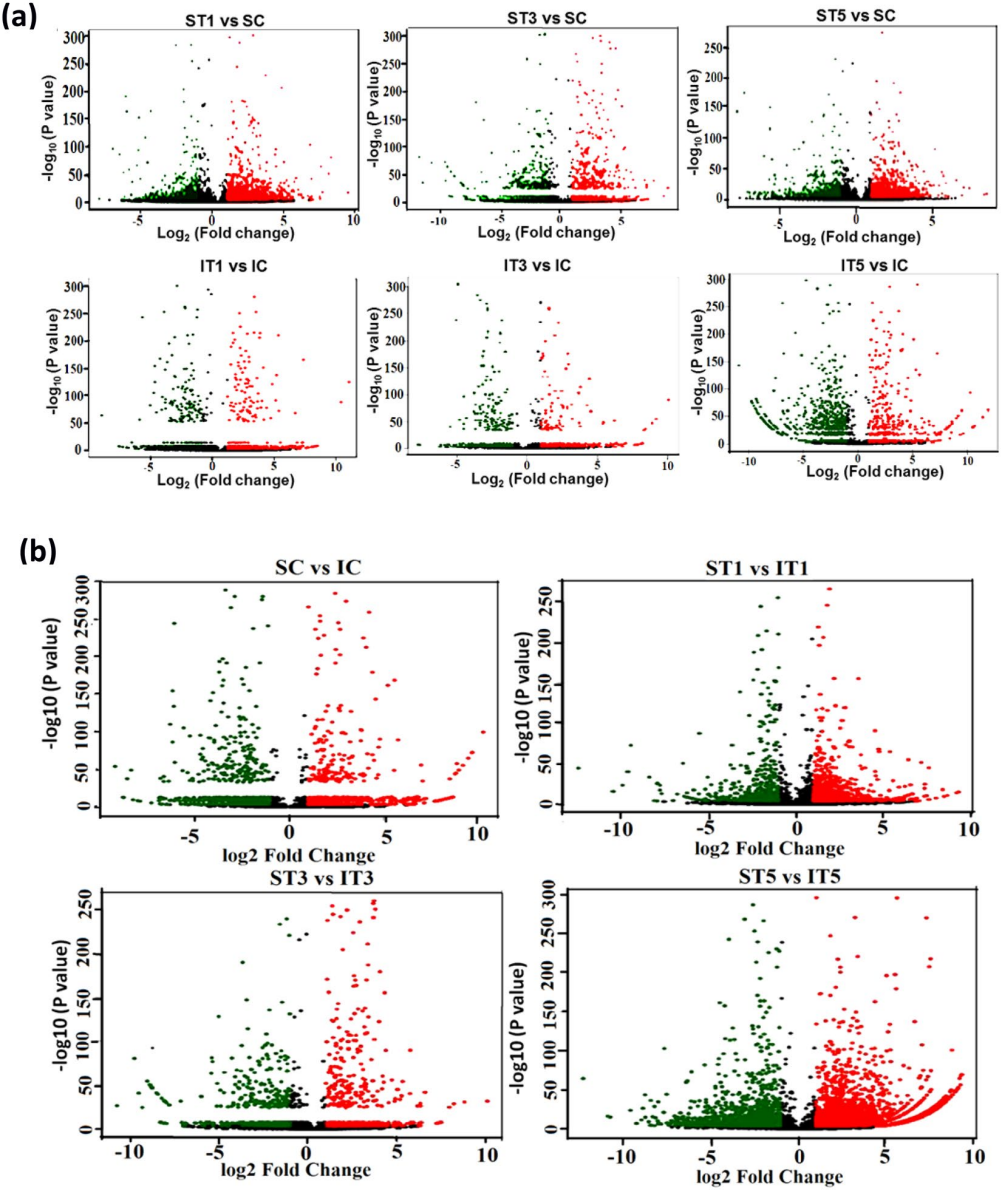
Volcano plot for differentially expressed genes. The x-axis shows the fold-change in gene expression between different samples, and the y-axis shows statistical significance of the differences. Significantly Up and Down-regulated genes are filtered (|log_2_ (Fold Change)| > 1, P_adj_ < 0.05) and highlighted in red and green dots, respectively. Genes that are not differentially expressed are in black. IC-IR8 control; IT1-IR8 for 1 day low light treated; IT3-IR8 for 3 days low light treated; IT5-IR8 for 5 days low light treated; SC- Swarnaprabha control; ST1- Swarnaprabha for 1 day low light treated; ST3- Swarnaprabha for 3 days low light treated; ST5-Swarnaprabha for 5 days low light treated.

**Figure 5:**
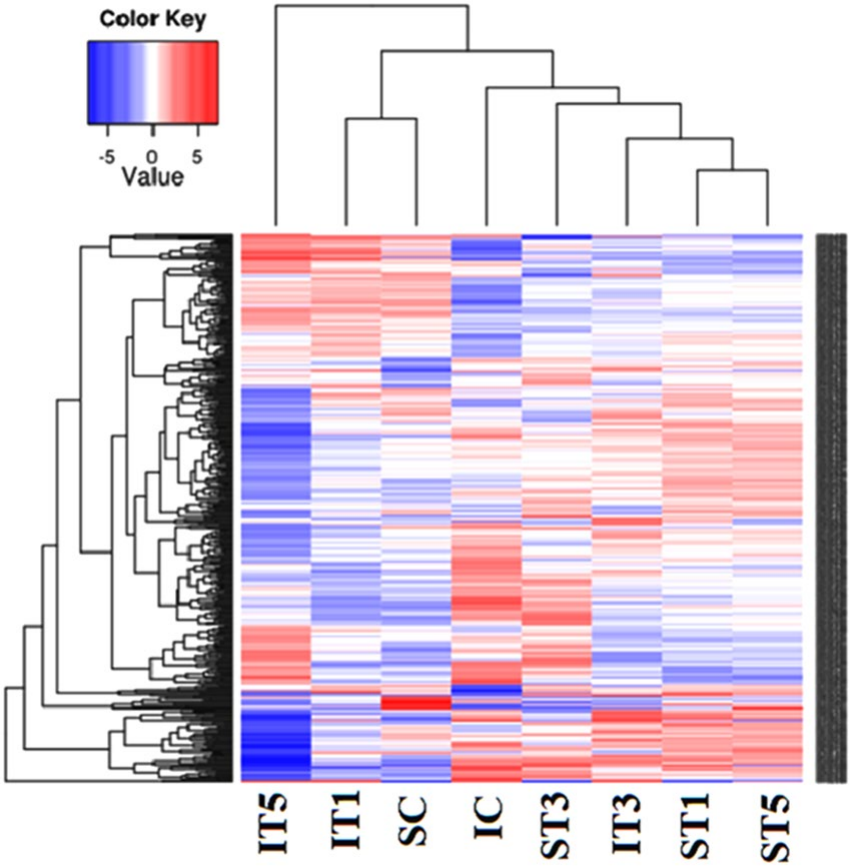
Heatmap of top 573 genes differentially expressed, blue colour represents low expressed genes and red colour represents highly expressed genes. Cluster analysis was done using the log_2_ (FPKM + 1) value from larger to smaller. IC-IR8 control; IT1-IR8 1 day low light treated; IT3-IR8 3 days low light treated; IT5-IR8 5 days low light treated; SC- Swarnaprabha control; ST1- Swarnaprabha 1 day low light treated; ST3- Swarnaprabha 3 days low light treated; ST5-Swarnaprabha 5 days low light treated.

Differential gene expression analysis was performed between control (C) and low light treated samples (T1, T3 and T5) for Swarnaprabha (low light tolerant) and IR8 (low light sensitive). The input data for differential gene expression analysis were read counts for the individual genes (Supplementary Table S2a and S2b). Correlation between samples is an important indicator for testing reliability of the experiment. Hence, Heat maps for the Pearson correlation coefficient between samples (Fig. 3) for differentially expressed genes were prepared, which showed correlation of the genes expressed in any two samples and closer the values of Pearson correlation coefficient to 1, greater the similarity between them in expression of genes. All samples except ST1 and ST5 showed greater difference in the expression of genes because of variety-specific and low light induced differences. To visualize the distribution of differentially expressed genes in between the samples (treated and control) within variety and in between varieties, volcano plots were generated (Fig. 4) in which x-axis shows the fold-change in expression of a gene and the y-axis shows statistical significance of the differences. Significantly up- and down-regulated genes were filtered and highlighted in red and green dots, respectively. Genes that were not differentially expressed were in black (Fig. 4a,b). Volcano plots in Fig. 4a shows distribution of differentially expressed genes between low light treated samples with respect to control within variety, whereas in Fig. 4b shows distribution of differentially expressed genes between varieties in control and under low light treated conditions. A |log_2_ (fold change)| > 1 and P_adj_ < 0.05, were used as standards for filtering the differentially expressed genes. Total number of differentially expressed genes, up-regulated and down-regulated genes in between varieties and within variety for treated and control samples were listed in Table 3 and details of their gene ids, read count and fold change were listed in Supplementary Table S1a–f. After filtering, a total of 6,652 and 12,042 genes were differentially expressed under low light treated condition in Swarnaprabha and IR8, respectively with respect to their control. A comparative analysis of treated samples with control revealed that 2,044 genes were differentially expressed between ST1 vs SC, among which 1,057 genes were up-regulated and 987 genes were down-regulated. However, between ST3 and SC, a total 5,884 genes were differentially expressed, among which 1,909 genes were up-regulated while 3,975 genes were down-regulated (Table 3). Between ST5 and SC, a total 1,975 differentially expressed genes, among which 1,059 genes were up-regulated and 916 genes were down-regulated. In IR8 samples, a total of 8,489 genes were differentially expressed between IT5 and IC, among which 2,989 genes were up-regulated and 5,500 genes were down-regulated. The differential expression between IT3 and IC was the next highest representing 5,986, among which 3,641 genes were up-regulated and 2,345 genes were down regulated upon low light treatment. The differential expression of the genes between IT1 and IC was more or less similar to that between IT3 vs IC representing 5,833 genes, among which 2,510 genes were up-regulated and 3,323 genes were down-regulated upon low light treatment. Further, when compared between both the varieties 1,823 genes were differentially expressed of which 1,163 were up-regulated and 660 were down-regulated in ST1 compared with that in IT1. However, upon increase in the duration of low light treatment, the number of genes differentially expressed between the two varieties increased. A total 1,748 genes were up-regulated and 3,729 genes were down-regulated between ST3 vs IT3. Similarly, between ST5 vs IT5, 4,906 genes were up-regulated and 2,121 genes were down-regulated. Furthermore, cluster of top 573 genes differentially expressed among samples are shown in the heat map (Fig. 5), which revealed that IT3, ST1 and ST5 are more similar than others in terms of the genes differentially expressed in response to low light treatment. Moreover, the number of genes up-regulated (Red colour) were more in ST1 and ST5 than others, while down-regulation of the genes were more pronounced in IT5 and IT1 than others.

**Table 3:** Number of up-regulated and down-regulated genes between low light treated and control samples of RNA sequencing results of rice variety Swarnaprabha and IR8 (P_adj_ < 0.05).

	Up-regulated	Down-regulated	Total
ST1 vs SC	1,057	987	2,044
ST3 vs SC	1,909	3,675	5,584
ST5 vs SC	1,059	916	1,975
IT1 vs IC	2,510	3,323	5,833
IT3 vs IC	3,641	2,345	5,986
IT5 vs IC	2,989	5,500	8,489
SC vs IC	3,927	2,848	6,775
ST1 vs IT1	1,163	660	1,823
ST3 vs IT3	1,748	3,729	5,477
ST5 vs IT5	4,906	2,121	7,027

SC- Swarnaprabha, control; ST1- Swarnaprabha for 1 day low light treated; ST3- Swarnaprabha for 3 days low light treated; ST5- Swarnaprabha for 5 days low light treated; IC- IR8, control; IT1- IR8 for 1 day low light treated; IT3- IR8 for 3 days low light treated; IT5- IR8 for 5 days low light treated sample.

**Figure 6:**
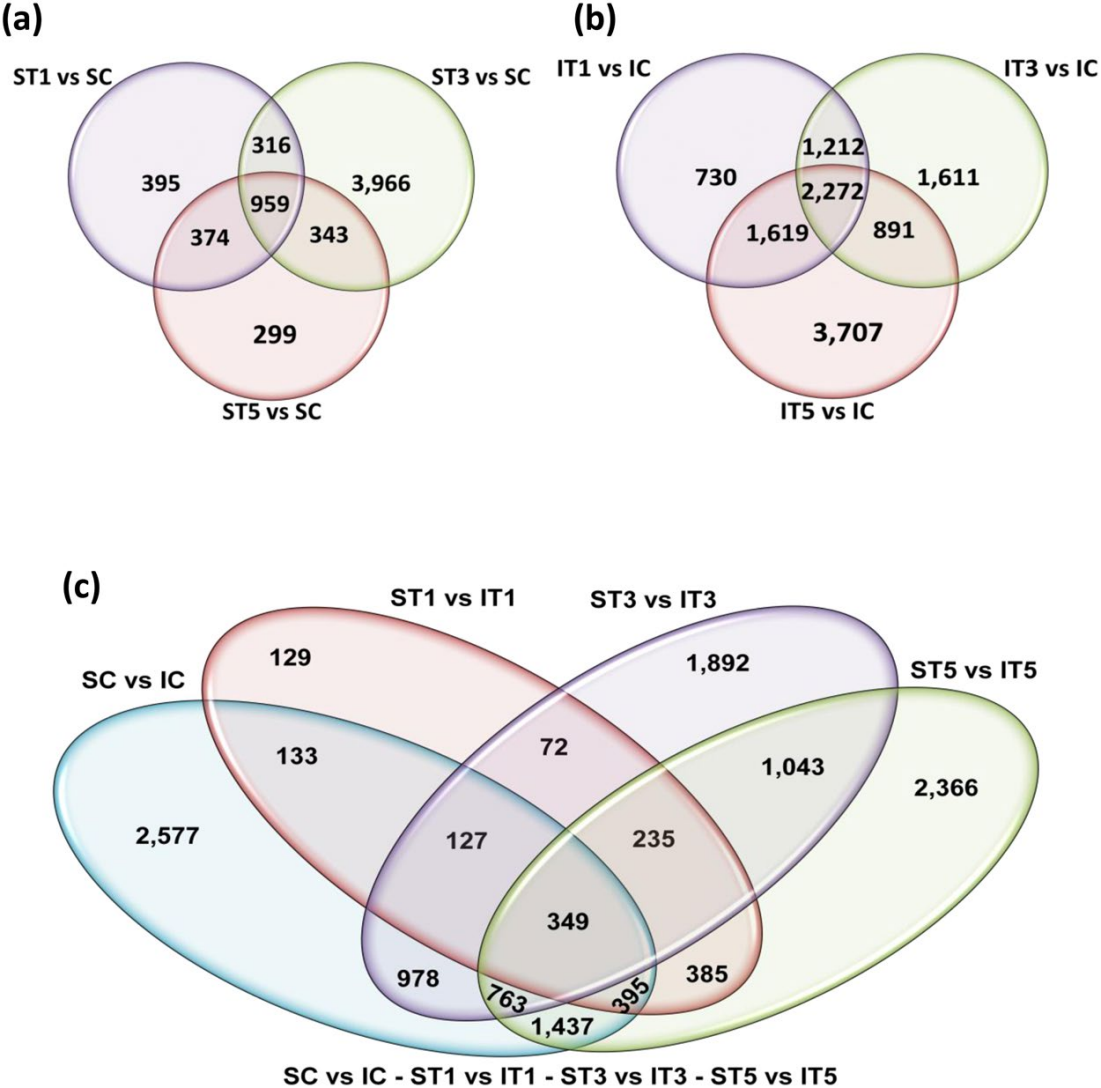
Venn diagram for differentially expressed genes between different comparisons of treated and control samples of Swarnaprabha and IR8. The sum of numbers in each circle is total number of differentially expressed transcripts within a group and the over-lap represents the genes expressed common in between comparisons. (**a**) Distribution of differentially expressed genes between ST1, ST3 and ST5 with respect to control. (**b**) Distribution of differentially expressed genes between IT1, IT3 and IT5 with respect to control. (**c**) Distribution of differentially expressed genes between SC, ST1, ST3, ST5 and IC, IT1, IT3, IT5. IC-IR8 control; IT1-IR8 for 1 day low light treated; IT3-IR8 for 3 days low light treated; IT5-IR8 for 5 days low light treated; SC- Swarnaprabha control; ST1- Swarnaprabha for 1 day low light treated; ST3- Swarnaprabha for 3 days low light treated; ST5-Swarnaprabha for 5 days low light treated.

Venn diagram analysis of distribution of the differentially expressed genes between control and low light treated samples (Fig. 6a,b) and between varieties under different low light treatment conditions revealed a complex scenario (Fig. 6c). Only 959 genes differentially expressed in response to low light treatment were found to be common in the Swarnaprabha (Fig. 6a) with respect to control. In contrast, the number of common differentially expressed genes in response to low light in IR8 was 2,272 (Fig. 6b) when compared to control. Moreover, 5 days low light treated sample of Swarnaprabha showed the least number of differentially expressed genes compared with the other day’s treatments, whereas the number of the differentially expressed genes in IR8, 5 days low light treated sample showed the maximum number of differentially expressed genes compared to the other treatments. The uncommon genes that expressed differentially in response to low light treatment were higher between the varieties (Fig. 6c). Out of several thousand genes that expressed differentially in response to low light treatment, only 349 genes were common between the two varieties in all treatments. The least uncommon differentially expressed genes were between ST1 vs IT1, followed by that between ST3 vs IT3, ST5 vs IT5, and SC vs IC. Thus, the differential expression of genes was much more in the low light sensitive IR8 than in the low light tolerant Swarnaprabha under low light stress.

### Gene Ontology and KEGG pathway analysis

GO enrichment and KEGG pathway enrichment analysis were carried out for the genes expressed differentially in response to low light compared to the control in both Swarnaprabha and IR8. Analysis was done between varieties and within variety compared with respective control. GO enrichment analysis of down-regulated and up-regulated genes was classified into three categories, including cellular components, biological processes and molecular functions (Supplementary Table S3a–t, Supplementary Fig. 3a–h). The genes involved in intracellular, cell part, organelle, intracellular organelle, cytoplasm, cytoplasmic and intracellular-vesicle, membrane-bound vesicle, plastid and mitochondrial related genes were predominant in cellular component. In the category of biological processes, the genes enriched were those involved in metabolic process, cellular process, cellular metabolic process, primary metabolic process, organic substance metabolic process, single-organism cellular process, cellular macromolecule metabolic process, protein metabolic process, nitrogen compound metabolic process, biosynthetic process, oxidation-reduction process. Similarly, the genes related to catalytic activity, binding, transferase activity, nucleic acid binding, small molecule binding, metal ion binding, carbohydrate derivative binding, ion binding, ATP binding, oxido-reductase activity, hydrolase activity, protein binding were enriched in the molecular function category. All the categories included both the genes getting down-regulated and the genes getting up-regulated. However, the number of up-regulated and down-regulated genes varied depending upon the varieties and the duration of exposure to low light. The number of genes up-regulated was more than those down-regulated when the comparison was made between SC vs IC. However, upon low light stress for 1 and 3 days, i.e. when the comparison was made between ST1 vs IT1 and between ST3 vs IT3, the number of genes that down-regulated increased significantly compared with those up-regulated. Upon 5 days low light exposure the pattern of expression however, changed again and more of the genes were up-regulated than down-regulated between ST5 vs IT5 (Supplementary Table S3g–h). KOBAS (KEGG Orthology Based Annotation System) was used for KEGG enrichment pathway analysis. A total of 561, 845 and 530 genes were differentially expressed in samples ST1, ST3 and ST5, respectively in pathway enrichment analysis. Among these genes that expressed differentially compared with control, 291 genes were down-regulated and 270 genes were up-regulated in ST1, 525 genes were down regulated and 320 genes were up-regulated in ST3 while 312 genes were down-regulated and 218 genes were up-regulated in ST5. The genes related to metabolic pathway, biosynthesis of secondary metabolite, ribosome, protein processing pathway, carbon catabolism, spliceosome and starch and sucrose metabolism pathway were mostly among those that differentially expressed in Swarnaprabha in response to low light exposure. In IR8, the differentially expressed genes in response to low light treatment were related to metabolic pathways, biosynthesis of secondary metabolites, ribosome, carbon metabolism, protein processing pathway, spliceosome, plant hormone signal transduction, photosynthesis, antenna protein and carbon fixation in photosynthesis (Supplementary Table S4a–t). Data analysis revealed that the differentially expressed genes in IR8 under low light exposure were more than double compared with that in Swarnaprabha with respect to control. Moreover, the genes related to photosynthesis pathway, carbon fixation pathway in photosynthesis and antenna protein were affected more in IR8 compared with that in Swarnaprabha.

### Expression analysis of differentially expressed genes by RT-PCR and qRT-PCR

Based on the read counts of the differentially expressed genes and their regulation under low light in photosynthesis related pathways, a few of them were selected for expression analysis by RT-PCR and further by qRT-PCR. Primers for each of these genes were designed and listed in Supplementary Table S5. Each cDNA preparation was normalized using actin as an internal control. Photosynthetic yield under low light stress depends entirely upon the efficiency of light energy capture by antenna pigments and its delivery to the reaction centers^17^. The light-harvesting complex (LHC) has chlorophyll a-b binding protein (CAB) as part of the antenna complex and functions as a light receptor that receives and transfer excitation energy to photosystem II and I^18^. Besides, the expression of CAB is regulated by many environmental factors, including light^19–22^. Six differentially expressed transcript for CAB picked from RNA sequencing data were selected for their expression analysis through RT-PCR and further through qRT-PCR.

**Figure 7:**
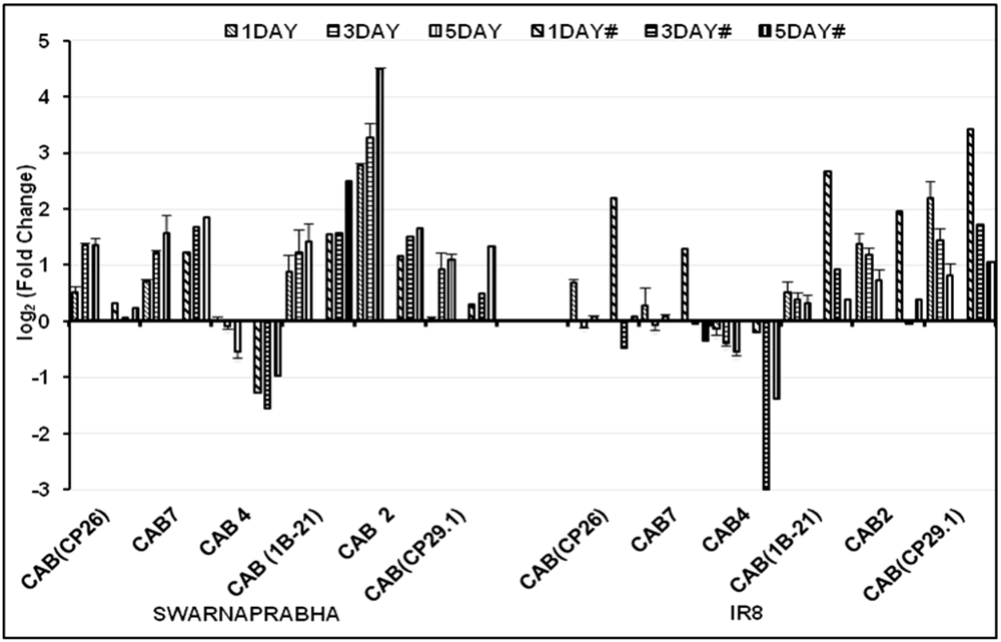
Validation of RNA-seq results of six isoforms of chlorophyll a-b binding protein (CAB) genes through qRT-PCR. Each gene was differentially expressed in low light tolerant (Swarnaprabha) and sensitive (IR8) rice varieties with respect to control and amplified with gene-specific primers designed using Primer Blast. Actin was taken as internal positive control. The primer sequences are provided in Supplementary Table S5. (# = RNA-seq); CAB, Chlorophyll a-b binding protein. Error bars are ± SD of the means of three qRT-PCR replicates.

**Figure 8:**
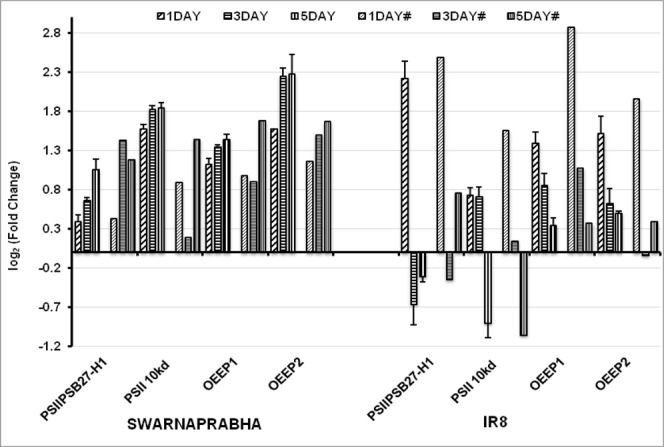
Validation of RNA-sequencing results through qRT-PCR of four differentially expressed genes involved in photosynthesis related pathway of low light treated sample of Swarnaprabha and IR8 with respect to control. Each gene was amplified using gene-specific primers designed using Primer Blast. Actin was taken as internal positive control. The primer sequences are provided in Supplementary Table S5. (# = RNA-seq). PSIIPSB27-H1, photosystem II repair protein PSB27-H1; PSII 10 kd polypeptide, photosystem II 10 kDa polypeptide; OEEP1, oxygen-evolving enhancer protein 1; OEE2, oxygen-evolving enhancer protein 2. Error bars are ±SD of the means of three qRT-PCR replicates.

**Figure 9:**
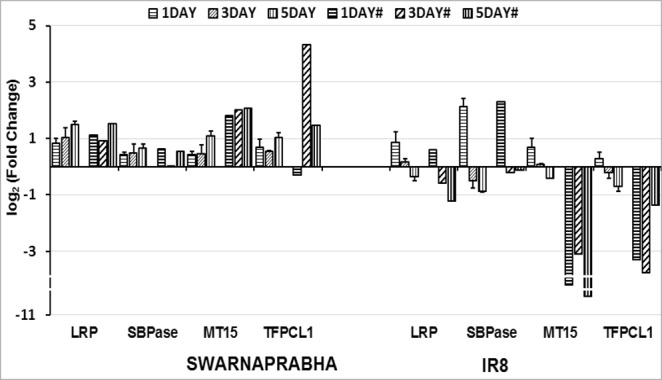
Validation of further four differentially expressed genes through qRT-PCR, selected from RNA sequencing results of Swarnaprabha and IR8 after low light treatment with respect to control. Each gene was amplified using gene-specific primers designed using Primer Blast and actin was taken as internal positive control. The primer sequences are provided in Supplementary Table S5. (# = RNA-seq). LRP, Light regulated protein, SBPase, sedoheptulose-1, 7-bisphosphatase, MT15, Plant metallothionein family 15 protein, TF PCL1, transcription factor PCL1. Error bars are ±SD of the means of three qRT-PCR replicates.

Expression analysis of CAB (CP26), CAB7, CAB (1B-21), CAB2 and CAB (CP29.1) by RT-PCR (Supplementary Fig. 2A) and further by qRT-PCR (Fig. 7) in the leaves of Swarnaprabha revealed that their expressions were up-regulated upon low light exposure compared with control. These genes showed expression pattern similar to that in RNA sequencing data. However, the expressions of CAB (CP26), CAB7, CAB (1B-21), CAB2 and CAB (CP29.1) were up-regulated upon 1 day low light exposure compared with control, but their expression was diminished after 1 day treatment in IR8. The expression of CAB4 was down-regulated in both the varieties similar to that found in RNA-sequencing data (Fig. 7). Thus, the qRT-PCR data revealed that increased expression of most of CAB in Swarnaprabha might be playing an important role in adaptation to low light stress, which was not observed in IR8. Four genes involved in metabolic pathway were validated for their expression through RT-PCR and qRT-PCR. One of them was chloroplastic gene (PSIIPSB27-H1) involved in energy metabolism of photosynthesis (subunit in electron transport of PS II). Temporal expression of PSIIPSB27-H1 upon exposure to low light stress with respect to control showed that its expression was up-regulated in all the treatments in Swarnaprabha, but its expression was up-regulated in one day low light treatment and then down-regulated upon increasing the treatment days in IR8 (Fig. 8). The expression of another protein, photosystem II 10 kDa polypeptide (PSII 10 kd polypeptide) was up-regulated in both the varieties except that upon five days exposure to low light stress, its expression was down-regulated in IR8. PSII 10 kd polypeptide remains associated with the oxygen-evolving complex of photosystem II. Other two proteins that remain associated with PSII are oxygen-evolving enhancer protein 1 (OEE1) and oxygen-evolving enhancer protein2 (OEE2). The expressions of both the genes were up-regulated in both the varieties after low light treatment except that in IR8, the expression of OEEP1 decreased from 1 day to 5 days with respect to control. All above data showed that assembly of photosystem II related genes were disturbed upon low light stress. Furthermore, expression of four genes, including light regulated protein (LRP), sedoheptulose-1, 7-bisphosphatase (SBPase), plant metallothionein family 15 protein (MT15) and transcription factor PCL1 (TF PCL1) were also analyzed through RT-PCR and qRT-PCR (Fig. 9). It was observed that their expressions were up-regulated in Swarnaprabha under all the low light treatments, but their expression was mostly down-regulated except after 1 day low light treatment in IR8.

## Discussion

Low light stress has adverse effect on plant growth and development, impairing several metabolic activities severely both at the active tillering and reproductive stages. Low light treatment was given to both the varieties for 1 day, 3 days and 5 days because during rainy season (Kharif season) in eastern India, cloudy conditions (low light) prevails not only for an hour or day but even also for 2 to 5 days that affects the tiller formation and its growth during vegetative stage in rice. Low light exposure for one day or less have least effect on photosynthetic process as it overcome the photosynthetic product in both tolerant and sensitive rice varieties but after longer duration of low light treatment i.e. for 3 to 5 days, sensitive varieties do not overcome with respect to tolerant as we have seen our transcriptome data that most of photosynthetic related genes were either increases or upregulated after one day low light treatment in both tolerant and sensitive rice varieties but after longer duration of low light treatment (3 to 5 days) the expression of genes were maintained or upregulated in low light tolerant variety but downregulated or decreased in low light sensitive variety which leads to significant loss in grain yield in low light sensitive rice varieties. Further photosynthetic active radiation (PAR) whose values varies and depends on the exposure of light intensity were recorded above canopy for confirmation of low light and normal light treatment in our experiment. PAR has linear correlation with the total dry matter production in rice^23–25^. Due to low light intensity treatment, a fraction of red light having range between 600–700 nm was decreased^26^ while blue light fraction having range between 400–500 nm was increased that affect the content of chlorophyll^27^, plant morphology like tiller appearance^28^ and physiological parameter like stomatal conductance^29–31^. In our experiment we have found that the chlorophyll a content was significantly increased in both the varieties under low light condition whereas chlorophyll b content was significantly increased in Swarnaprabha therefore the chlorophyll a/b ratio was significantly decreased in Swarnaprabha. However, chlorophyll b content was not significantly increased in IR8, shows that low light tolerant variety Swarnaprabha increases its chlorophyll b content to greater extent under low light condition. Similar results were also reported in wheat in low tolerant cultivar of wheat Yangmai 158 and low light sensitive wheat cultivar like Yangmai 11^32^. Further in our experiment we have found that physiological parameters like stomatal conductance, transpiration and net assimilation values increased in tolerant variety Swarnaprabha as compared to sensitive variety IR8, under the condition of low light treatment. Stomatal conductance and net photosynthesis has positive correlation^33^, it means increased physiological parameter play positive impact to enhance the net photosynthesis under low light condition in low light tolerant varieties Swarnaprabha. Plant system has mechanism to lower the effect of stress through temporal expression of the related genes. Transcriptome profiling upon low light stress in rice has not been studied yet. In this study, comparative analysis of transcriptome of two rice varieties, Swarnaprabha (low light tolerant) and IR8 (low light sensitive) was carried out. The transcriptome analysis revealed significant difference between them. The differentially expressed transcripts (Supplementary Table S1a–f) in response to low light stress might have collective role in maintaining the normal metabolic activities in the plants under low light condition, particularly that driven by light. Some of differentially expressed genes known for their role in light driven metabolism were considered for their expression analysis through RT-PCR and qRT-PCR. Genes encoding Chlorophyll a-b binding proteins (CAB), an apo-protein of the light-harvesting complex of photosystem II (PSII) are normally complexed with chlorophyll and xanthophyll^34,35^. It works as an antenna complex, which absorb sunlight and transfer the excitation energy to drive photosynthetic electron transport chain. The expression of CAB is regulated by multiple environmental factors, and light intensity is one of them^36^. Expression and abundance of CAB consistently changes in response to light intensity^37,38^. The amount of LHCII is increased for adaptation to low light stress, as it enhances the efficiency of light harvesting by PSII^39^. In our experiment, it was observed that most of the CAB expression was up-regulated after 1 day low light stress. But, their expressions decreased in IR8 compared to control, upon subsequent days of low light treatment. However, their expressions remained greater than control even after 3 days and 5 days of low light exposure in Swarnaprabha. The results indicated the important role of the CAB proteins for adaptation to low light condition, which occurs in Swarnaprabha but not in IR8. The reason for the down-regulation of temporal expression of CAB4 in both the varieties in response to low light treatment is unclear. Similar to CAB proteins, up-regulation of PSIIPSB27-H1 (a component of PSII complex) in Swarnaprabha and its down-regulation in IR8 (except on 1 day low light treatment) with respect to control gives a clue for its important role in adaptation to low light stress in rice, although any role of PSIIPSB27-H1 in low light stress protection has not been reported till date. Nevertheless, it is well established that it has role in energy metabolism of photosynthesis and in repair of photo damaged photosystem II^40^. Earlier, it is also reported that PSB27 protein facilitates manganese cluster assembly in photosystem II^41^. Low light tolerant variety exhibits higher amount of light absorbing pigment molecules like chlorophyll b and lower chlorophyll a/b ratio facilitating capture of light energy under low light to drive the transfer of electrons from water to plastoquinone which is true from the present investigation^10^. In this context, the increased expressions of PSII 10 KD polypeptide, OEE1 and OEE2 associated with Photosystem II complex in Swarnaprabha upon exposure to low light from 1 day onwards compared to control, suggested their protective role under low light condition for enabling the plant to keep light reaction of photosynthesis at same pace even under low light condition to fulfill the downstream process of PSII. Up-regulation of expression of four genes, namely, light regulated protein (LRP), sedoheptulose-1 transcription factor (SBPase), plant metallothionein family 15 protein (MT15) and transcription factor PCL1 (TF PCL1) in Swarnaprabha under low light stress could also be an important mechanism of tolerance to the stress. This is due to the presence of light regulated protein (LRP) or light-induced rice1 (LIR1) that interact with leaf-type ferredoxin- NADP^+^ oxidoreductase (LFNR), which is an essential chloroplast enzyme functioning in the last step of photosynthetic linear electron transfer^42^. LFNR catalyzes electron transfer from ferredoxin (FD) to NADP+ during photosynthesis, which generates the reducing power as NADPH that is used in carbon fixation^43^. It is also report that regulation of LFNR between stroma and chloroplast membrane is fine-tuned by light through the action of Light regulated protein in rice and *Arabidopsis*^42^. In our study, increase in expression of LRP in Swarnaprabha and decreased expression in IR8 compared to control (except 1 day low light treatment case) under low light stress showed that Swarnaprabha might be able to maintain the formation of NADPH used in carbon fixation through more interaction of LRP with LFNR. Sedoheptulose-1, 7-bisphosphatase (SBPase) also has very important role in photosynthetic carbon fixation in the Calvin cycle, but its expression has not been studied under low light condition till date. Up-regulation of SBPase in Swarnaprabha and its down-regulation in IR8 under low light condition indicated that former could be more efficiently driving the Calvin cycle than the latter under low light stress. However, unlike LRP and SBPase, the role of plant metallothionein family15 protein (MT15) in plant metabolism, particularly its involvement in photosynthesis is totally unknown. Plant metallothioneins (MTs) are a family of cysteine-rich, a low molecular weight and metal-binding proteins. They play important role in hormone treatment, detoxification of heavy metal ions and osmotic stresses^44,45^. The present investigation also indicated their possible important role in light reaction of photosynthesis as the expression of MT15 was up-regulated in Swarnaprabha but down-regulated in IR8 upon low light exposure. Transcription factor PCL1, a myb family transcription factor unique to plants is essential for circadian rhythms. It encodes a novel DNA binding protein belonging to the GARP protein family and is essential for regulating the circadian clock^46^. In higher plants, the circadian clock regulates many processes, including photoperiodic flowering induction, leaf movement, hypocotyl elongation, photosynthetic activity, etc^46–48^. The possible role of PCL1 in low light acclimation in plants is not known. Nevertheless, its reported involvement in photosynthetic activity certainly indicates that it might have some regulatory role in adaptation of plants to low light. Up-regulation of its expression in Swarnaprabha and down-regulation in IR8 after low light treatment for longer period of exposure compared to control also indicates the involvement of PCL1 in this process. The expression of the gene was up-regulated in one day low light stress in both the varieties, but afterward, the expression of PCL1 was down-regulated in IR8 indicating that in initial stage both the varieties tried to adapt to low light but only Swarnaprabha could maintain its expression for longer time necessary for adaptation. Overall, it is possible that many factors might be involved in regulation of gene expression under low light stress for combating the slowing down of the light driven metabolism. The above genes that expressed differentially and were mostly upregulated in Swarnaprabha upon low light treatment might be the factors facilitating the plants to continue their vegetative and reproductive growth unhindered even at low light intensity.

The present work on transcriptome profiling and analysis of the genes differentially expressed in tolerant Swarnaprabha and susceptible IR8 varieties provided a comprehensive insight into the genes involved in low light toleranance in rice. The study clearly indicated that the expression of certain genes were significantly up-regulated under low light in the tolerant variety Swarnaprabha, but were down-regulated in the sensitive variety IR8, indicating their probable inolvement in low light tolerance. The details of the tolerance mechanism are however, yet to be understood, although the biochemical and molecular funtions of most of them are known. These genes constitute as good candidate genes to understand the signaling mechanism of low light tolerance in rice that could be considered for improvement of low light tolerance in the variety of interest through biotechnological interventions.

## Materials and Methods

### Plant material and treatment

Two contrasting rice varieties for low light tolerance and sensitive, for example Swarnaprabha (low light tolerant) and IR8 (low light sensitive), were grown in the plant physiology net house of ICAR-National Rice Research Institute (NRRI), Cuttack, Odisha, India. Low light intensity (LL) were exposed using Agro shade net (50% of normal light) during vegetative stage (active tillering stage), 40 days after germination. Three treatments were maintained with T1 for 1 day, T3 for 3 days and T5 for 5 days under low light stress. Normal light intensity (NL) exposure was maintained for control plants. Leaves were sampled from the main tillers of five different plants at the end of the low light stress exposure with control plant. The samples collected were first frozen in liquid nitrogen and then stored at −80 °C until use.

### Measurement of Photosynthetic active radiation (PAR)

Spatiotemporal distribution of PAR above the canopy of plants under low light and normal light conditions for both the varieties, Swarnaprabha and IR8 were recorded using radiometer (LI-1500 LICOR, USA) thrice per day (9.00 am, 12.00 pm and 4.00 pm of Indian Standard Time (IST), UTC + 05:30). Five replicas under each condition were maintained for accurate PAR measurement.

### Chlorophyll content and Photosynthetic parameter calculation

Chlorophyll a and b content of both normal light (NL) and low light (LL) samples of Swarnaprabha and IR8 were estimated. Flag leaves from each replicate were taken, mid rib removed, sliced and sample extracted with 10 ml 80% acetone^49^. The extracts were stored in the dark for 24 hr and then the absorbance was recorded at 663 nm and 645 nm using the colorimetric method to estimate the chlorophyll a and b contents in mg per gram fresh weight (mg g^−1^ fw)^50^. Five replicates were used for chlorophyll a and b estimation.

The net photosynthetic rate (Pn) of the flag leaf was determined using a LI-6400XT portable photosynthesis system (LI-COR, Inc., USA) for both Swarnaprabha and IR8 under normal and low light conditions. Net assimilation, stomatal conductance and transpiration measurements were recorded for both open system and treatment with a CO_2_ concentration of 380 µmol l^−1^ under available light condition^50^. An average value was calculated from five flag leaves from each replicate.

### Total RNA extraction

Total RNA was isolated from the control and treated samples using TRIZOL reagent (Invitrogen) following the manufacturer’s instruction. Individual leaf samples were collected from five different plants, mixed together and total RNA was extracted. The individual RNA pellets obtained were suspended in DEPC treated water and the concentration of total RNA was measured using Qubit 2.0 Fluorometer. Quality checking of each sample was performed with Qubit (picogreen). The RNA samples were stored at −80 °C until use.

### Library construction and transcriptome sequencing

A total of eight libraries were prepared, four for Swarnaprabha (named as ST1- 1 day, ST3- 3 days, ST5- 5 days low light treated and SC- control) and four for IR8 (named as IT1- 1 day, IT3- 3 days, IT5- 5 days low light treated and IC- control). For library preparation, individual sample was collected from five different plants and mixed for total RNA isolation and subjected to mRNA enrichment. This was done with the help of Ribo-Zero kit. The poly-A containing mRNA molecules from total RNA in the individual sample were pulled out using oligo-dT attached magnetic beads. Following this, the mRNA was fragmented into small pieces using divalent cations under elevated temperature. The cleaved RNA fragments were used as template for the synthesis of first strand cDNA using reverse transcriptase and random primers. Second strand cDNA synthesis was done using DNA polymerase I and RNase H. The cDNA fragments were then subjected to an end repair process, the addition of a single ‘A’ base, and ligated adapters. The products were then enriched using PCR to create the final cDNA library for each sample for Sequencing. The quality controls (QC) passed libraries was performed on next generation sequencing (NGS) Illumina HiSeq 2500 platform using Paired end (PE) 2 × 150 bp sequencing approach. The HiSeq 2500 System, a powerful and efficient ultra-high-throughput sequencing system. The base calling pipeline - HiSeq Control Software 2.2.38, RTA 1.18.61.0, CASAVA-1.8.2 was used for sequencing.

### Bioinformatics analysis of sequencing data

Raw sequence data obtained for the treated and control samples were analyzed using In-house script for pre-processing of raw reads. Low quality and adapter contaminated reads were filtered. FastQC tools were used for quality control. Clean reads from each sample were aligned individually to *Oryza sativa japonica (Ensembl IRGSP-1*.*0)* reference genome using TopHat 2^51^. TopHat is a fast splice junction mapper for RNA-Sequence reads. It aligns RNA-Sequence reads to large size genomes using the ultrahigh-throughput short read aligner Bowtie 2^52^, and then analyzes the mapping results to identify splice junctions between exons. HTSeq tool used to count the number of reads aligned to protein coding genes. HTSeq is a Python package that provides infrastructure to process data from high-throughput sequencing assays.

### Differential gene expression analysis

Differential gene expression analysis was carried out for each sample with respect to control and between samples of tolerant variety Swarnaprabha and sensitive variety IR8. The input data for differential gene expression analysis were read counts generated from sequencing and bioinformatics processing of the raw reads. The differential gene expression analysis included three steps: Read counts normalization, model dependent p-value estimation, FDR (false discovery rate) value estimation based on multiple hypothesis testing. Trimmed mean of M values (TMM) were used for normalization, DEGseq and HTseq^53,54^ software were used for filtering the sequences and p value were adjusted for multiple testing with the Benjamini-Hochberg procedure which controls false discovery rate (FDR). The Gene Ontology and KEGG enrichment analysis were done by using KOBAS^55^ for understanding the function of the genes and their involvement in various biological processes. The genes with Q-value 0.005 were used for KEGG enrichment analysis. KOBAS can identify enriched pathways and functional terms for an input set of genes using biological knowledge from well-known pathway databases. Hyper geometric test/Fisher’s exact statistical test method was used for enrichment analysis while Benjamini and Hochberg test was used for FDR correction.

### Gene expression analysis by RT-PCR and qRT-PCR

For validation of the next generation sequencing (NGS) results on the differentially expressed genes, total RNA extracted from the frozen samples using TRIZOL reagent was used for cDNA preparation following the standard protocol^56^. The primers with Tm 58 °C to 62 °C and a length of 19-mer to 22-mer were designed for the gene of interest using the Primer Blast software at the NCBI site (http://www.ncbi.nlm.nih.gov/tools/primer-blast/). The primers designed were specific to *O*. *sativa* and amplified fragments ranging from 100 to 250 bp (Supplementary Table S5). Prior to designing the primer, the individual sequence of the *japonica* cultivar group was searched for similarity with the *indica* cultivar group at the Ensemble Plants site (http://plants.ensembl.org/index.html). RT-PCR and qRT-PCR were performed by same method, reagent and instrument as mentioned in the standard method^56^.

## Supplementary information

Supplementary information

Supplementary table S 1a-f

Supplementary table S 2a and S 2b

Supplementary table S 3a-t

Supplementary table S 4a-t

Supplementary Table S5
