# Supplementary material for: Comparative transcriptome profiling of low light tolerant and sensitive rice varieties induced by low light stress at active tillering stage
Source: Sci Rep. 2019 Apr 8;9:5753. doi: 10.1038/s41598-019-42170-5 (PMC6453891; doi:10.1038/s41598-019-42170-5)
Supplement: Supplementary file 1 — Supplementary information [file 41598_2019_42170_MOESM1_ESM.pdf]

**Comparative transcriptome profiling of low light tolerant and sensitive rice varieties induced by low light stress at active tillering stage**

Sudhanshu Sekhar[1], Darshan Panda[1], Jitendra Kumar[1], Niharika Mohanty[1], Monalisha Biswal[1], Mirza J. Baig[1], Awadhesh Kumar[1], Ngangkham Umakanta[1], Sangamitra Samantaray[1], Sharat K. Pradhan[1], Birendra P. Shaw[2], Padmini Swain[1], Lambodar Behera[1*]

**Supplementary information:**

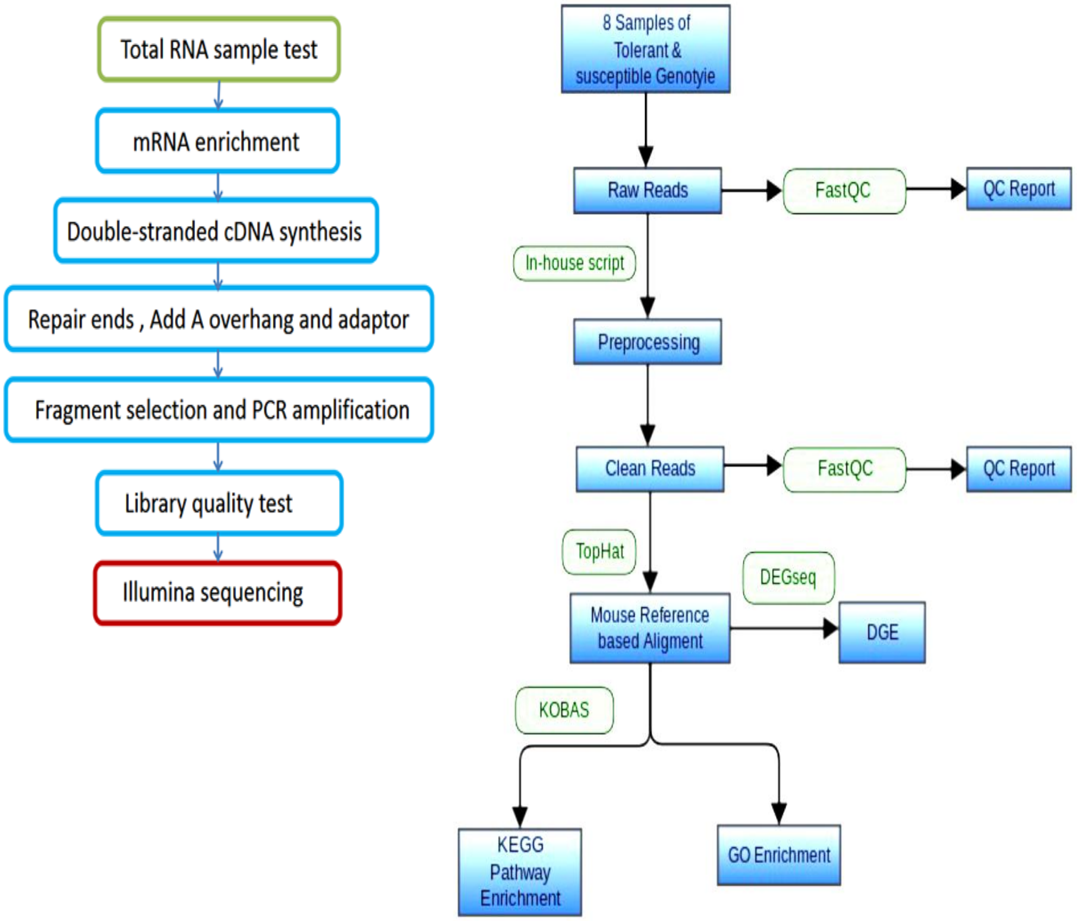


**Supplementary Figure S1.** Workflow of Illumina sequencing and bioinformatics analysis carried out for the rice sample of low light treated and control for both the cultivar Swarnaprabha and IR8.

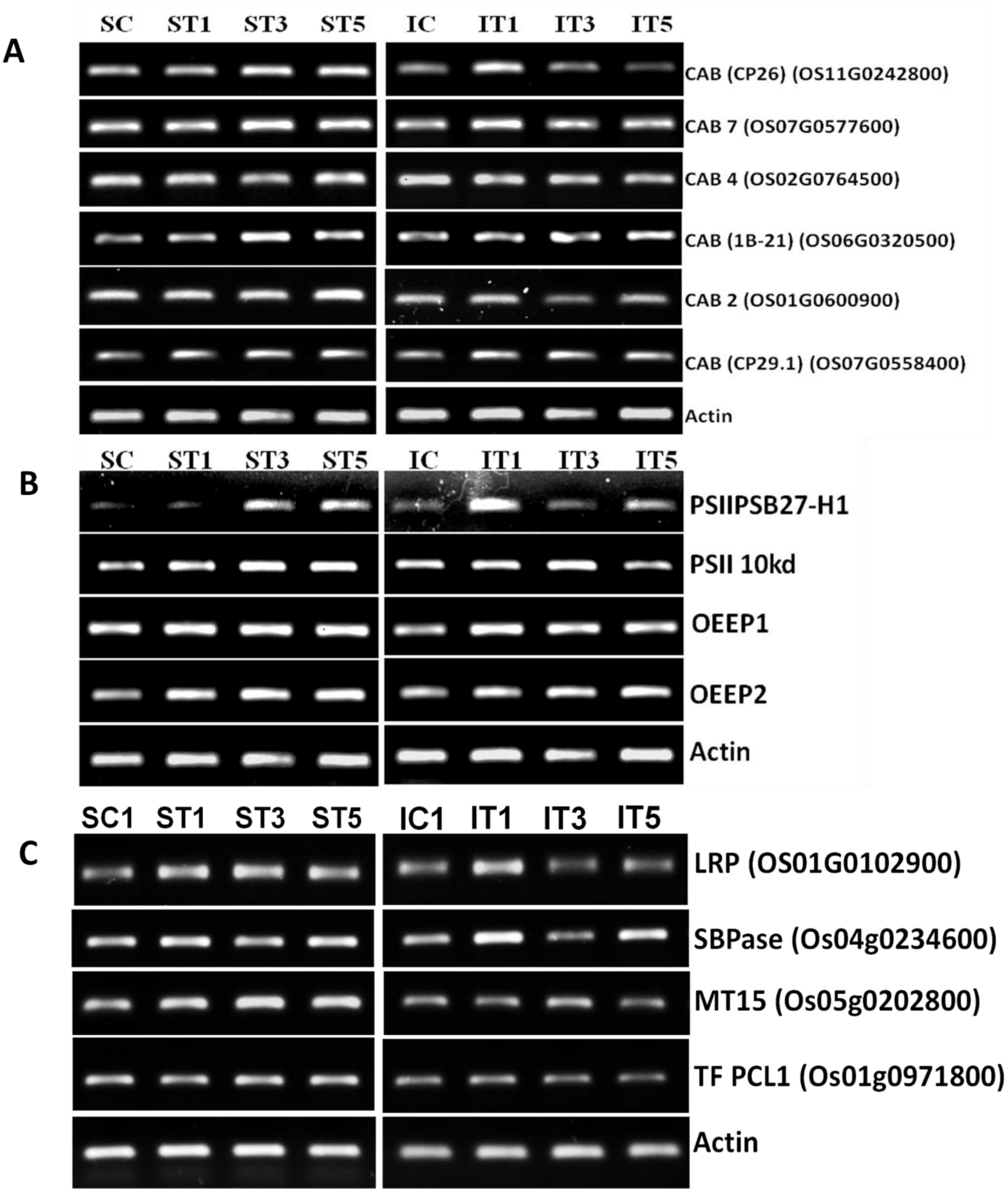

A
SC
ST1
ST3
ST5
IC
IT1
IT3
IT5
CAB (CP26) (OS11G0242800)
CAB 7 (OS07G0577600)
CAB 4 (OS02G0764500)
CAB (1B-21) (OS06G0320500)
CAB 2 (OS01G0600900)
CAB (CP29.1) (OS07G0558400)
Actin
B
SC
ST1
ST3
ST5
IC
IT1
IT3
IT5
PSIIPSB27-H1
PSII 10kd
OEEP1
OEEP2
Actin
C
SC1
ST1
ST3
ST5
IC1
IT1
IT3
IT5
LRP (OS01G0102900)
SBPase (Os04g0234600)
MT15 (Os05g0202800)
TF PCL1 (Os01g0971800)
Actin

**Supplementary Figure S2.** RT-PCR data for differentially expressed (NGS transcriptome data) between and within cultivar after low light treatment. Each gene was amplified using gene-specific primers designed using Primer Blast and actin was amplified for 30 cycles, respectively as positive controls. The amplified products were separated on an agarose gel containing ETBR and visualized and photographed using a Gel Doc (Bio-Rad). The primer sequences are provided in Supplementary Table S5. SC- Swarnaprabha control, ST1- Swarnaprabha treated for 1day under low light; ST3- Swarnaprabha treated for 3days under low light; ST5- Swarnaprabha treated for 5days under low light; IC- IR8 control; IT1- IR8 treated for 1day under low light; IT3- IR8 treated for 3days under low light; IT5- IR8 treated for 5 days under low light. CAB- Chlorophyll a-b bonding protein; PSIIPSB27-H1, photosystem II repair protein PSB27-H1, PSII 10kd polypeptide, photosystem II 10 kDa polypeptide, OEEP1, oxygen-evolving enhancer protein 1, OEE2, oxygen-evolving enhancer protein 2; LRP, Light regulated protein; SBPase, sedoheptulose-1, 7-bisphosphatase; MT15, Plant metallothionein family 15 protein; TF PCL1, transcription factor PCL.

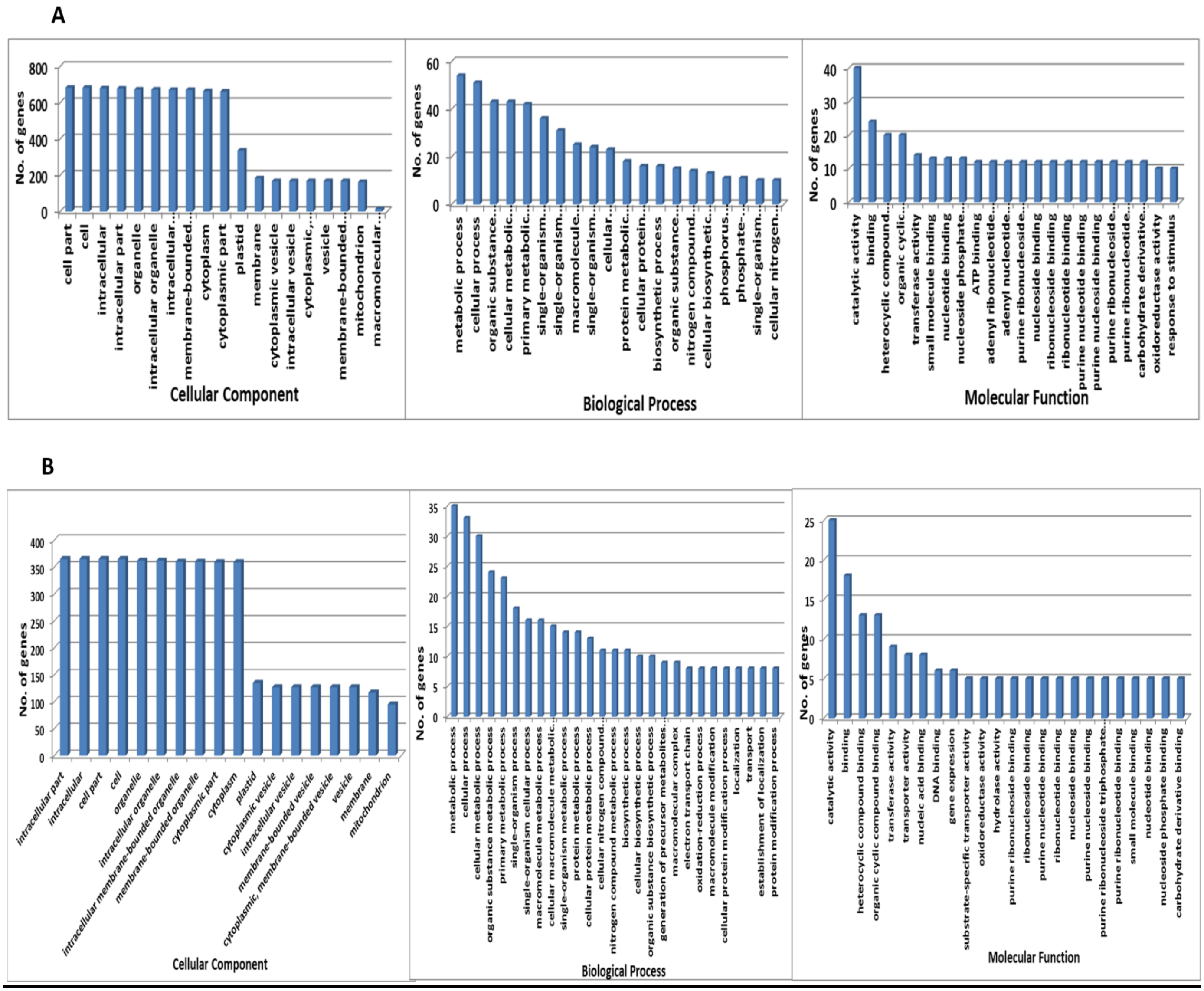

A
No. of genes
800
600
400
200
0
cell part
cell
intracellular
intracellular part
organelle
intracellular organelle
intracellular...
membrane-bounded...
cytoplasm
cytoplasmic part
plastid
membrane
cytoplasmic vesicle
intracellular vesicle
cytoplasmic,...
vesicle
membrane-bounded...
mitochondrion
macromolecular...
Cellular Component
No. of genes
60
40
20
0
metabolic process
cellular process
organic substance...
cellular metabolic...
primary metabolic...
single-organism...
single-organism...
macromolecule...
single-organism...
cellular...
protein metabolic...
cellular protein...
biosynthetic process
organic substance...
nitrogen compound...
cellular biosynthetic...
phosphorus...
phosphate-...
single-organism...
cellular nitrogen...
Biological Process
No. of genes
40
30
20
10
0
catalytic activity
binding
heterocyclic compound...
organic cyclic...
transferase activity
small molecule binding
nucleotide binding
nucleoside phosphate...
ATP binding
adenyl ribonucleotide...
adenyl nucleotide...
purine ribonucleoside...
nucleoside binding
ribonucleoside binding
ribonucleotide binding
purine nucleotide binding
purine nucleoside binding
purine ribonucleoside...
purine ribonucleotide...
carbohydrate derivative...
oxidoreductase activity
response to stimulus
Molecular Function
B
No. of genes
400
350
300
250
200
150
100
50
0
intracellular part
intracellular
cell part
cell
organelle
intracellular organelle
intracellular membrane-bounded organelle
membrane-bounded organelle
cytoplasmic part
cytoplasm
plastid
cytoplasmic vesicle
intracellular vesicle
membrane-bounded vesicle
cytoplasmic, membrane-bounded vesicle
vesicle
membrane
mitochondrion
Cellular Component
No. of genes
35
30
25
20
15
10
5
0
metabolic process
cellular process
cellular metabolic process
organic substance metabolic process
primary metabolic process
single-organism process
single-organism cellular process
macromolecule metabolic process
cellular macromolecule metabolic...
single-organism metabolic process
protein metabolic process
cellular protein metabolic process
cellular nitrogen compound...
nitrogen compound metabolic process
biosynthetic process
cellular biosynthetic process
organic substance biosynthetic process
generation of precursor metabolites...
macromolecular complex
electron transport chain
oxidation-reduction process
macromolecule modification
cellular protein modification process
localization
transport
establishment of localization
protein modification process
Biological Process
No. of genes
25
20
15
10
5
0
catalytic activity
binding
heterocyclic compound binding
organic cyclic compound binding
transferase activity
transporter activity
nucleic acid binding
DNA binding
gene expression
substrate-specific transporter activity
oxidoreductase activity
hydrolase activity
purine ribonucleoside binding
ribonucleoside binding
purine nucleotide binding
ribonucleotide binding
nucleoside binding
purine nucleoside binding
purine ribonucleoside triphosphate...
purine ribonucleotide binding
small molecule binding
nucleotide binding
nucleoside phosphate binding
carbohydrate derivative binding
Molecular Function

C

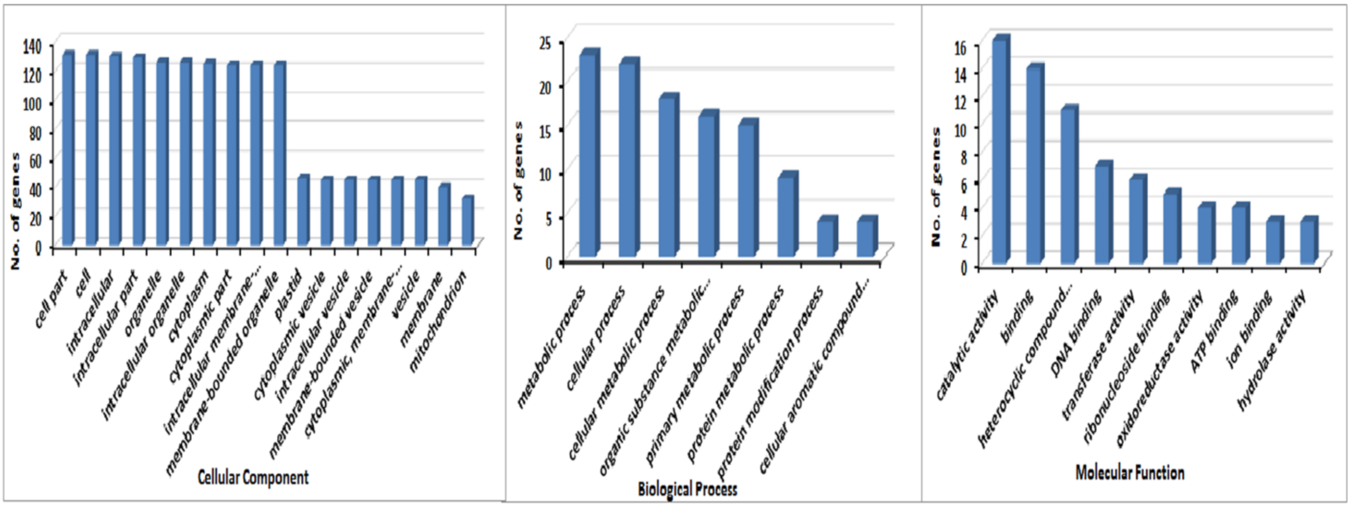

No. of genes
Cellular Component
cell part
cell
intracellular
intracellular part
organelle
intracellular organelle
cytoplasm
cytoplasmic part
intracellular membrane-...
membrane-bounded organelle
plastid
cytoplasmic vesicle
intracellular vesicle
membrane-bounded vesicle
cytoplasmic, membrane-...
vesicle
membrane
mitochondrion
Biological Process
metabolic process
cellular process
cellular metabolic process
organic substance metabolic...
primary metabolic process
protein metabolic process
protein modification process
cellular aromatic compound...
Molecular Function
catalytic activity
binding
heterocyclic compound...
DNA binding
transferase activity
ribonucleoside binding
oxidoreductase activity
ATP binding
ion binding
hydrolase activity


D

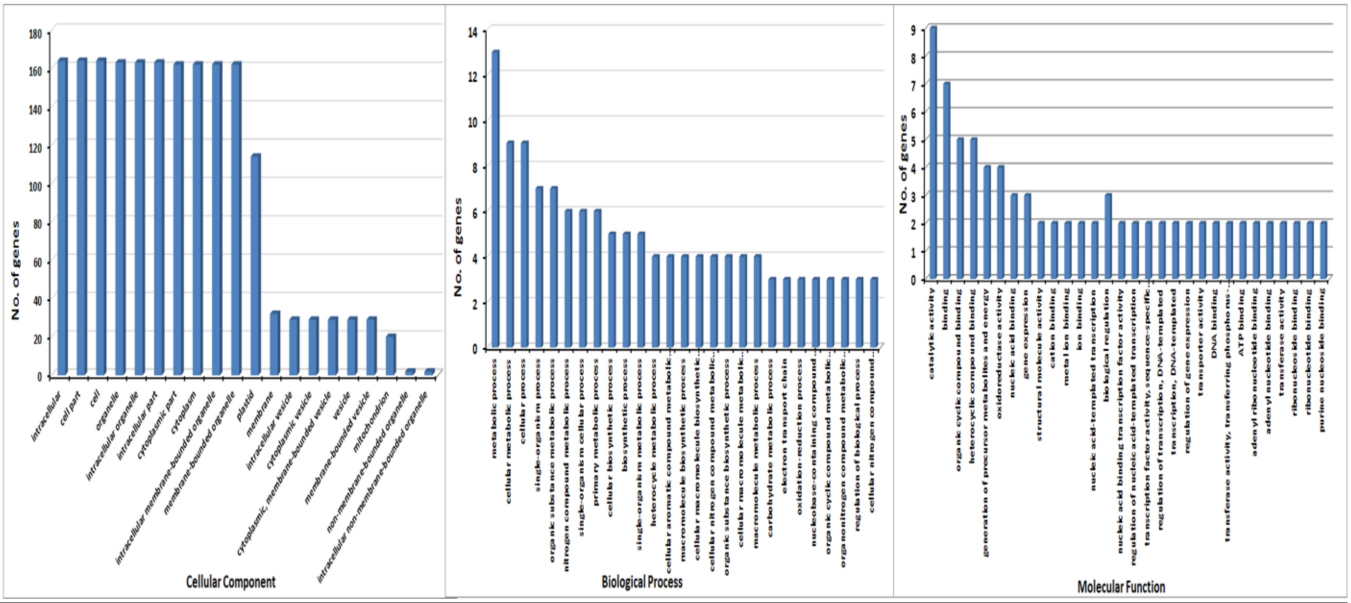

No. of genes
Cellular Component
Biological Process
Molecular Function

E

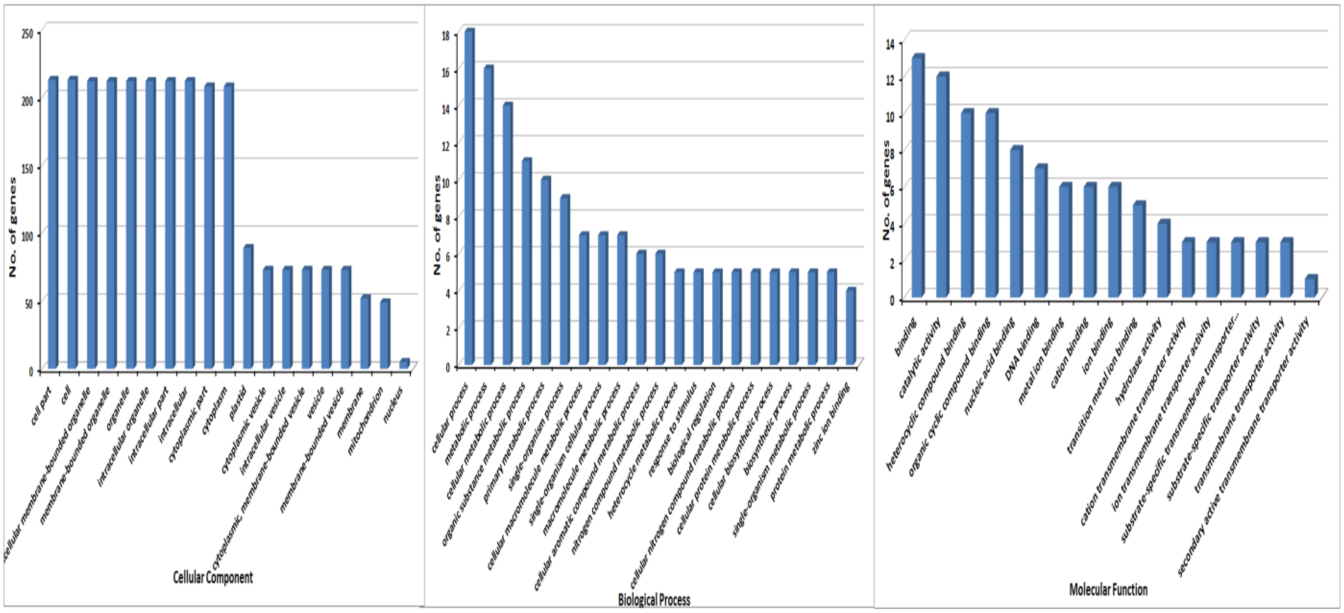

No. of genes
Cellular Component
cell part
cell
cellular membrane-bounded organelle
membrane-bounded organelle
organelle
intracellular organelle
intracellular part
intracellular
cytoplasmic part
cytoplasm
plastid
cytoplasmic vesicle
intracellular vesicle
cytoplasmic, membrane-bounded vesicle
vesicle
membrane-bounded vesicle
membrane
mitochondrion
nucleus
Biological Process
cellular process
metabolic process
cellular metabolic process
organic substance metabolic process
primary metabolic process
single-organism process
cellular macromolecule metabolic process
single-organism cellular process
macromolecule metabolic process
cellular aromatic compound metabolic process
nitrogen compound metabolic process
heterocycle metabolic process
response to stimulus
biological regulation
cellular nitrogen compound metabolic process
cellular protein metabolic process
cellular biosynthetic process
biosynthetic process
single-organism metabolic process
protein metabolic process
zinc ion binding
Molecular Function
binding
catalytic activity
heterocyclic compound binding
organic cyclic compound binding
nucleic acid binding
DNA binding
metal ion binding
cation binding
ion binding
transition metal ion binding
hydrolase activity
cation transmembrane transporter activity
ion transmembrane transporter activity
substrate-specific transmembrane transporter...
substrate-specific transporter activity
transmembrane transporter activity
secondary active transmembrane transporter activity


F

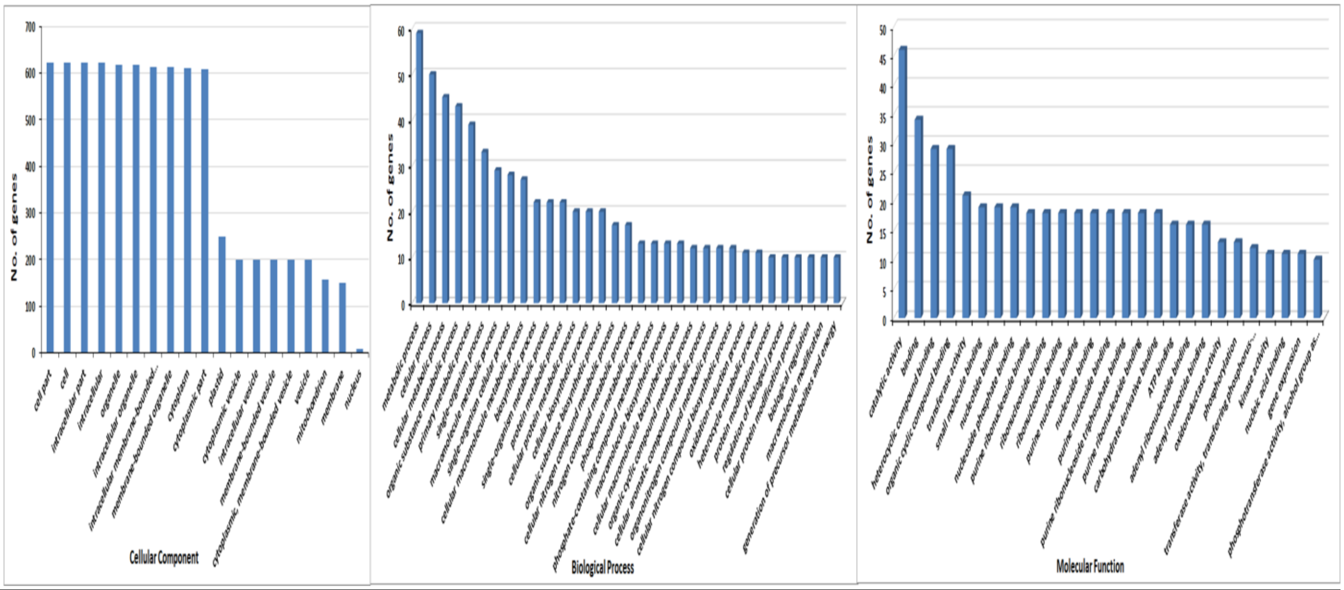

No. of genes
Cellular Component
Biological Process
Molecular Function

G

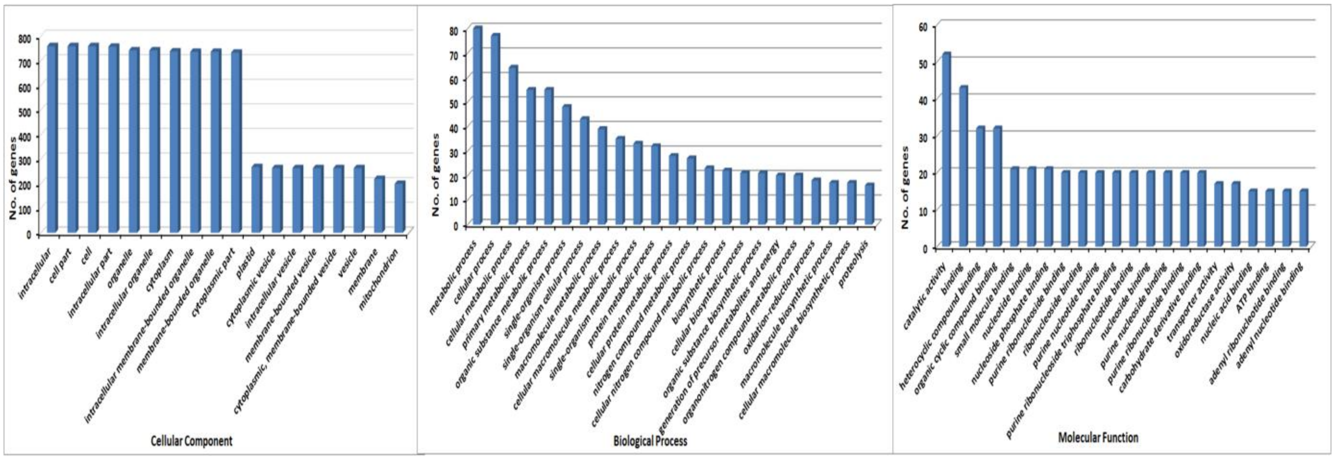


H

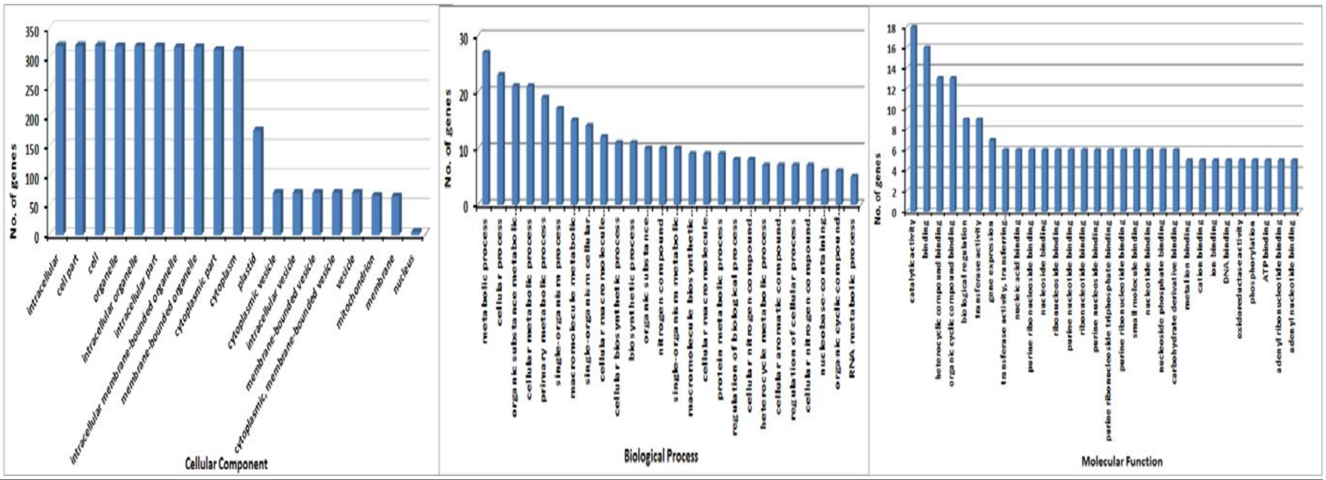


**Supplementary Figure S3 A-H. Go analysis of differentially expressed gene in cellular component, biological process and molecular function of Control and low light treated sample, Comparative analysis was done within control, treated and control and in between treated sample of Swarnaprabha and IR8 with different time point. (A) SC vs IC Up-regulated genes (B) SC vs IC down-regulated genes (C) ST1 vs IT1 up-regulated genes (D) ST1 vs IT1 Down-regulated genes (E) ST3 vs IT3 up-regulated genes (F) ST3 vs IT3 down-regulated genes (G) ST5 vs IT5 up-regulated genes (H) ST5 vs IT5 Down-regulated genes.**
